# Molecular characterization and analysis of the drug resistance-associated protein phosphoglycerate kinase of *Eimeria tenella*

**DOI:** 10.1017/S0031182024001355

**Published:** 2024-10

**Authors:** Yu Yu, Wenhao Huang, Qiping Zhao, Shunhai Zhu, Hui Dong, Hongyu Han

**Affiliations:** Chinese Academy of Agricultural Sciences, Key Laboratory of Animal Parasitology of Ministry of Agriculture, Shanghai Veterinary Research Institute, Minhang, Shanghai, PR China

**Keywords:** drug resistance, *Eimeria tenella*, phosphoglycerate kinase

## Abstract

Coccidiosis is a parasitic disease caused by *Eimeria* spp., and the emergence of drug resistance has seriously affected the control of the disease. Using RNA-seq, we previously found that phosphoglycerate kinase of *Eimeria tenella* (*Et*PGK) was differentially downregulated in diclazuril-resistant (DZR) and maduramicin-resistant (MRR) strains compared with drug-sensitive (DS) strain. In this study, we further analysed the characteristics and functions of *Et*PGK to find the possible mechanism of drug resistance of *E. tenella*. Quantitative real-time PCR (qRT-PCR) and western blot found that *Et*PGK was highly expressed in sporulated oocysts, followed by sporozoites and second-generation merozoites of *E. tenella*. Indirect immunofluorescence localization showed that *Et*PGK was located mainly in the cytoplasm and on the surface of the parasites. Invasion inhibition assays showed that anti-r*Et*PGK antibody significantly inhibited the invasion of parasites. Further studies using qRT-PCR and western blot found that the transcription and translation levels of *Et*PGK were downregulated in both resistant (DZR and MRR) strains compared with the DS strain, and the transcription level correlated negatively with the drug concentration. The enzyme activity assay revealed that *Et*PGK enzyme activity was decreased in the DZR strain compared with the DS strain. qRT-PCR revealed that the mRNA transcription level of *Et*PGK was significantly downregulated in the field DZR strain and salinomycin-resistant strain compared with the DS strain. These results suggested that *Et*PGK has other important roles that are separate and distinct from its function in glycolysis, and it might be involved in the development of drug resistance of *E. tenella*.

## Graphical abstract

**Figure:**
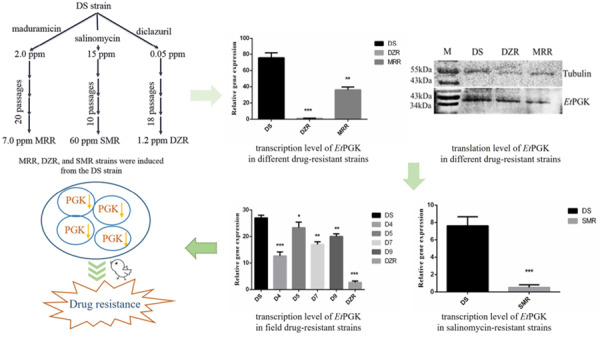

## Introduction

Chicken coccidiosis is a serious protozoan disease caused by *Eimeria* spp. parasitizing the intestines. It is estimated that the global poultry industry incurs losses of up to $14 billion annually due to coccidiosis (Blake *et al*., 2020). Even low doses of *Eimeria* oocysts can cause intestinal pathology, alter intestinal morphology and impair intestinal barrier function and development (Gelinas *et al*., 2024). *Eimeria* infection is widely recognized as a primary risk factor for necrotic enteritis caused by *Clostridium perfringens* (Blake *et al*., 2021). In addition, infection with *Eimeria* spp. leads to intestinal flora imbalance, which affects the structure and diversity of microbial communities as well as the transmission of foodborne zoonotic pathogens, resulting in losses that are difficult to quantify.

For a long time, the large-scale use of anti-coccidial drugs has been an efficient method for controlling coccidiosis (Peek and Landman, 2011). Two main types of anti-coccidial drugs are currently used: chemically synthesized drugs (including diclazuril and decoquinate) and polyether ionophores (including maduramicin and salinomycin). Maduramicin and diclazuril are widely used to control coccidiosis with remarkable efficacy. Maduramicin interferes with the permeability of the coccidia cell membrane to affect the monovalent cations. The coccidia use energy to exclude the large amount of Na^+^ entering the cell, and finally, the coccidia die due to energy depletion (Kant *et al*., 2013). Diclazuril affects the nucleic acid synthesis of coccidia and prevents the differentiation of schizonts and microgametocytes (McDougald *et al*., 1990; Hunyadi *et al*., 2015). However, the widespread use of drugs over time has created a severe problem of drug resistance. Since the isolation of sulfonamide-resistant strains from chickens on farms in the United States in 1954, strains resistant to almost all anticoccidial drugs have been isolated. Furthermore, multiple cross-resistant strains have appeared (Abbas *et al*., 2011; Zhang *et al*., 2023). Drug resistance has become the most important obstacle in the prevention and control of coccidiosis.

To study the molecular mechanism of drug resistance, our laboratory successfully induced and obtained diclazuril-resistant (DZR) and maduramicin-resistant (MRR) strains based on a drug-sensitive (DS) strain of *Eimeria tenella* using a drug concentration-increasing method. The obtained DZR strain was fully resistant to 1.2 ppm diclazuril, while the MRR strain was only fully resistant to 7.0 ppm maduramicin (Han *et al*., 2004). We then used RNA-sequencing to screen genes differentially expressed among the DZR, MRR and DS strains of the same genetic background. The results showed differential expression of phosphoglycerate kinase of *E. tenella* (*Et*PGK), which was significantly downregulated in the DZR and MRR strains compared with the DS strain (Xie *et al*., 2020).

Phosphoglycerate kinase (PGK) is an essential enzyme in the second phase of the aerobic glycolysis pathway. It catalyses the conversion of 1,3-bisphosphoglycerate to 3-phosphoglycerate, during which 1 molecule of ADP is consumed to produce 1 molecule of ATP. If the reverse reaction occurs, 1 molecule of ADP is formed (He *et al*., 2019). In addition to regulating cellular metabolism, PGK is involved in a wide range of biological activities, including DNA repair, autophagy and angiogenesis (Banks *et al*., 1979). Abnormal levels of PGK expression have been associated with disease development. For example, PGK deficiency is related to neurological damage, Parkinson's disease and myopathy (Echaniz-Laguna *et al*., 2019; Hogrel *et al*., 2019). PGK is a drug resistance-related protein in cancer cells, and high expression of PGK protein may promote drug resistance through complex molecular mechanisms (He *et al*., 2019). PGK has been studied in parasites such as *Plasmodium falciparum*, *Trypanosoma cruzi* and *Leishmania major* but has not been reported in *Eimeria*.

In this study, *Et*PGK was cloned and successfully expressed. We evaluated the differential expression of *Et*PGK in drug-resistant strains and the DS strain. In addition, we analysed the expression levels and distribution of *Et*PGK at different developmental stages of *E. tenella*.

## Materials and methods

### Animals and parasites

The yellow chickens used in the experiment were supplied by a farm (Shanghai, China), and the New Zealand rabbits were purchased from the Jiagan Biology Company (Shanghai, China). All animals were reared in a coccidia-free environment with free access to feed and water.

*Eimeria tenella* DS strain was isolated from a farm in Shanghai (Resource Number CAAS21111601) and kept in our laboratory, maintained by passage every 3 months in susceptible 14-day-old yellow-feathered chickens (Huang *et al*., 1993). Our laboratory-induced MRR, DZR and salinomycin-resistant (SMR) strains were each only resistant to 7.0 ppm maduramicin, 1.2 ppm diclazuril and 60 ppm salinomycin, respectively (Han *et al*., 2004; Wang *et al*., 2019). Three drug-resistant strains were also passaged by infected chickens feeding corresponding drugs.

The unsporulated oocysts (UO) were collected and purified using standard procedures, and sporulated oocysts (SO) were formed after oxidation of UO at 28°C (Han *et al*., 2010). Sporozoites (SZ) were collected from SO *in vitro* (Miska *et al*., 2004). The second-generation merozoites (SM) were collected from the cecum infected with *E. tenella* 120 h after inoculation (Zhou *et al*., 2010).

The single-oocyst method was used to isolate the field diclazuril-resistant strains (D4, D5, D7 and D9). Their resistance to 1.0 ppm diclazuril was demonstrated using drug sensitivity experiments in chickens (Khalafalla and Daugschies, 2010).

### Amplification, cloning and sequencing of *Et*PGK

Total RNA was extracted from SO of *E. tenella* DS strain using TRIzol reagent (TaKaRa, Tokyo, Japan). The RNA was treated with DNase I and transcribed into cDNA using a reverse transcriptase kit (Invitrogen, Carlsbad, CA, USA) and oligo (dT) primer. cDNA was then used as the amplification template.

The ORF of *Et*PGK (ToxoDB Accession No.: ETH_00015140) was amplified by PCR using the primers 5′-GCGGAATTCATGCGCGTGGACTTCAACGTGCCG-3′ (sense) and 5′-GCGCTCGAGATTCTCCAGGAGCTCCAGAGAG-3′ (antisense), including *EcoR* I and *Xho* I restriction sites. Amplified products were analysed and purified by 1% agarose gel electrophoresis (Qiagen, Dusseldorf, Germany) and subcloned into the pGEM-T-easy vector (Promega, Madison, WI, USA). The positive clone for recombinant plasmid was identified by DNA sequence.

The full-length cDNA sequence of *Et*PGK was analysed using BLAST program of the National Center for Biotechnology Information (https://www.ncbi.nlm.nih.gov/BLAST/). SignalP (https://www.cbs.dtu.dk/service s/SignalP) was used to predict signal peptide, Motif Scan (https://hits.isb-sib) was used to predict protein motifs, and the transmembrane motif was determined by TMHMM (https://www.cbs.dtu.dk/services/T MHMM-2.0/).

### Quantitative real-time PCR (qRT-PCR)

The mRNA expression of *Et*PGK at different developmental stages (UO, SO, SZ and SM) of *E. tenella* DS strain was determined by real-time quantitative PCR (qRT-PCR). The qRT-PCR was performed using SYBR1 Green I dye method. Total RNA from UO, SO, SZ and SM was extracted as described above, and genomic DNA was removed using RNeasy Mini Kits (Qiagen). cDNA was synthesized from total RNA of different developmental stages using SuperScript II reverse transcriptase (Invitrogen) and random primers (Invitrogen). The *Et*PGK primers used for qRT-PCR were 5ʹ-GCTGCTGACCTGGCTGCTGACCTGCTGCTGCT-3′ (sense) and 5ʹ-ACGTGCCGCTCAAAGACGGG-3′ (antisense). The 18S rRNA housekeeping gene (GenBank number: EF122251) of *E. tenella* was used as an internal control with primers 5ʹ-TGTAGTGGAGTCTTGTGGATTC-3′ (sense) and 5ʹ-CCTGCTGCC TTCCTTAGATG-3′ (antisense). Relative expression of *Et*PGK was measured using the 2-^ΔΔCt^ method (Livak and Schmittgen, 2001). Reactions were performed in triplicate and experiments were repeated 3 times.

We also compared the transcription levels of *Et*PGK in SO of DZR, MRR, SMR and DS strains using qRT-PCR. The transcription levels of DZR strains resistant to different concentrations of diclazuril (0.2, 0.5, 0.8 and 1.0 ppm) and MRR strains resistant to different concentrations of maduramicin (3 and 5 ppm) were analysed compared with DS strain. The transcription levels of *Et*PGK in the field DZR strains (D4, D5, D7 and D9) were also detected using qRT-PCR.

### Protein expression and polyclonal antibody preparation

The expression vector pGEX-4T-1 (Novagen, Darmstadt, Germany) and the recombinant plasmid pGEM-T-*Et*PGK were double-digested with *EcoR* I and *Xho* I, and then ligated to construct the recombinant expression plasmid pGEX-4 T-*Et*PGK. pGEX-4 T-*Et*PGK plasmid was transformed into *Escherichia coli* BL21 (DE3) (Tiangen, Beijing, China) and induced expressed recombinant protein (r*Et*PGK) with 1.0 mm isopropyl *β*-D-1-thiogalactopyranoside (IPTG; Sigma-Aldrich, St. Louis, MO, USA). The expression form of r*Et*PGK was determined by 12% sodium dodecyl sulphate polyacrylamide gel electrophoresis (SDS-PAGE) analysis after sonication. r*Et*PGK was purified from SDS-PAGE gel strips (Richard, 2009). The quality of purified r*Et*PGK was identified by 12% SDS-PAGE, and the concentration of purified protein was determined by BCA protein detection reagent (Beyotime, Haimen, China).

Three-month-old New Zealand rabbits were immunized subcutaneously with purified r*Et*PGK (200 *μ*g per rabbit) 5 times at 1-week intervals. Freund's complete adjuvant (Sigma-Aldrich) and r*Et*PGK protein were mixed at a ratio of 1:1 for the first immunization, and Freund's incomplete adjuvant was used for subsequent immunizations (Sigma-Aldrich). Polyclonal antibodies to r*Et*PGK were collected 7 days after the last immunization. Serum collected before protein injection was used as a negative control.

### Western blot

The expression levels of *E*tPGK in UO, SO, SZ and SM of *E. tenella* DS strain were analysed by western blot. Total proteins from 4 stages were prepared using cell lysis buffer. The concentration of protein was determined using a BCA protein assay kit (Beyotime). The same quantity of proteins (20 *μ*g) was separated by 12% SDS-PAGE and transferred to polyvinylidene difluoride membranes (Millipore, Burlington, MA, USA). The membranes were blocked with 5% (w/v) skimmed milk in phosphate-buffered saline (PBS). The primary antibody was anti-r*Et*PGK polyclonal rabbit antibody, while *α*-tubulin mouse antibody (Beyotime) was used as a control. HRP-conjugated goat anti-rabbit IgG or HRP-conjugated goat anti-mouse IgG (H + L) was used as secondary antibody. The bands were detected with a Supersignal West Pico Plus chemiluminescent substrate kit (Thermo Fisher, Waltham, MA, USA) using Odyssey Infrared Imaging System (LI-COR Biosciences, Lincoln, NE, USA).

Using the same method, the translation level in SO of *Et*PGK among DS, DZR and MRR strains was determined using r*Et*PGK-immunised rabbit serum and *α*-tubulin mouse antibody as primary antibody by western blot.

### Localization of *Et*PGK by indirect immunofluorescence

The freshly extracted SZ and SM were evenly applied to the glass slides and then placed in the 6-well plate to dry. The glass slides were placed into the 6-well plate and subsequently inoculated with DF-1 cells (3 × 10^5^ per well) and cultured for 24 h. Purified SZ (9 × 10^5^ cells per well) were added to invade and develop in the cells. The glass slides were removed at various time points after inoculation, then washed and air-dried. The cells were fixed in 4% paraformaldehyde solution for 30 min, permeabilized with 1% Triton X-100 for 15 min and then blocked with 2% (w/v) bovine serum albumin at 37°C for 2 h. Then the rabbit anti-r*Et*PGK was added and incubated at 37°C for 2 h. Slides were then incubated with goat anti-rabbit IgG fluorescein isothiocyanate-conjugated antibody (Sigma-Aldrich) at 37°C for 40 min. Nuclei of parasites and cells were stained with 10 mg mL^−1^ 40, 6-diamidino-2-phenylindole (Beyotime) for 15 min at room temperature. After each step, glass slides were washed 5 times with PBST. Slides were treated with 50 *μ*L Fluoromount Aqueous Mounting Medium (Sigma-Aldrich) and observed under a fluorescence microscope (Olympus, Tokyo, Japan).

### Sporozoite invasion inhibition assay

Referring to the method of Wilson *et al*. (2015), an invasion assay was performed to test the effect of rabbit anti-r*Et*PGK polyclonal antibody on the invasion of DF-1 cells by SZ. The 2 × 10^5^ DF-1 cells were added in advance to a 24-well plate and cultured for 24 h. Freshly extracted SZ of DS strain were labelled with carboxyfluorescein diacetate succinimidyl ester (Beyotime), then incubated with different concentrations of purified rabbit IgG against r*Et*PGK (50, 100, 200, 300 and 400 *μ*g mL^−1^), with the same concentration of normal rabbit serum IgG as a control, and a blank group was set at the same time. After incubation at 41°C for 2 h, SZ were centrifugated and resuspended in complete culture medium. Then SZ (6 × 10^5^ per well) were added to the 24-well plate containing DF-1 cells, and cultured for 6 h at 41°C and 5% CO_2_. Uninvaded SZ were washed by PBS, and the invasion rate of SZ into DF-1 cells was detected by flow cytometry.

### Enzyme activity

Whole proteins of fresh DS, DZR and MRR strains of SO (1 × 10^7^) were obtained by sonication on ice. The concentration of protein was determined using a BCA protein assay kit (Beyotime). The protein concentration was diluted to 0.5 mg mL^−1^ with RNAase-free water and determined spectrophotometrically using the *Et*PGK Enzyme Activity Assay Kit (Thermo Scientific). Then, 50 *μ*L of reaction buffer was added to each well and mixed with the substrate, and 50 *μ*L of 0.5 mg mL^−1^ DS, DZR or MRR protein was added. The results were observed after 20 min at 340 nm.

## Results

### Characterization of the *Et*PGK sequence

**Figure 1.**
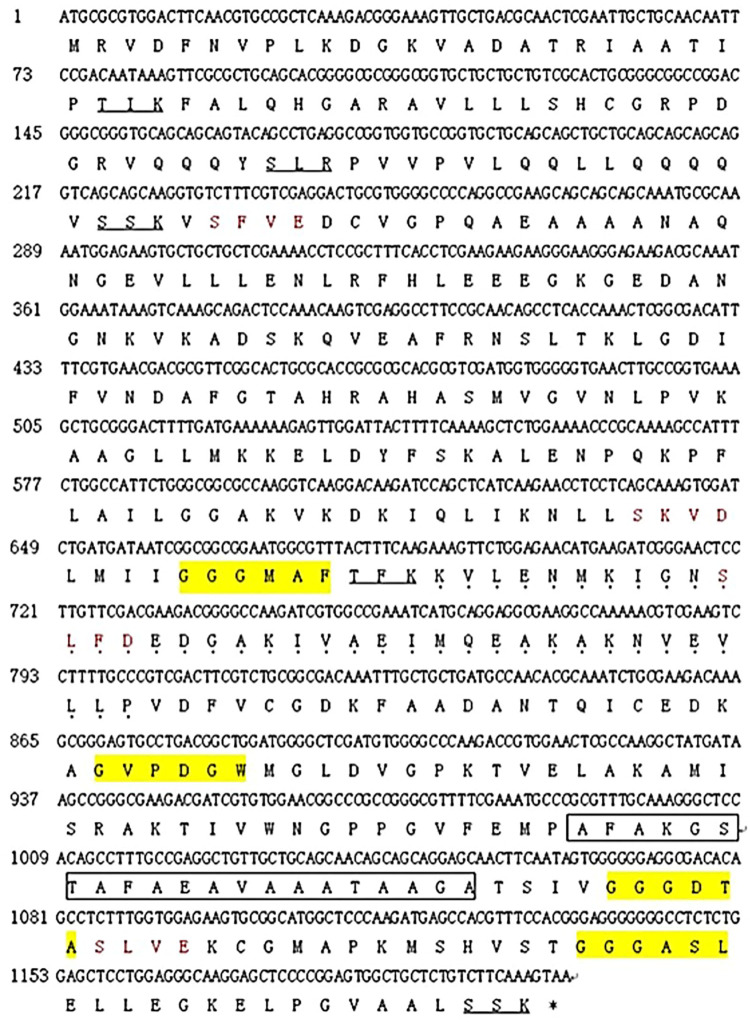
Bioinformatic analysis of *Et*PGK. Black spots: Pumilio RNA-binding repeat profile; box: alanine-rich region profile; underline: protein kinase C phosphorylation site; yellow: N-myristoylation site; red: casein kinase II phosphorylation site; *stop codon.

The ORF sequence of *Et*PGK we amplified was 100% homologous to *E. tenella* phosphoglycerate kinase (GenBank: XM_013373443.1) and 91% homologous to *Eimeria necatrix* phosphoglycerate kinase (GenBank: XM_013373443.1). Analysis of the *Et*PGK nucleotide sequence showed that the *Et*PGK sequence was 1206 bp, encoding 401 amino acid residues, with an isoelectric point of 7.01 and a predicted molecular weight of 42.3 kDa. There was no signal peptide or transmembrane region. The protein encoded by this gene contained 4 casein kinase II phosphorylation sites (78–81, 213–216, 240–243, 362–365); 4 N-myristoylation sites (221–226, 290–295, 356–361, 379–384); 5 protein kinases C phosphorylation sites (26–28, 56–58, 74–76, 227–229, 399–401); an alanine-rich region (331–351); and a Pumilio RNA-binding repeat sequence (230–267) (Fig. 1).

### Expression and purification of recombinant *Et*PGK

**Figure 2.**
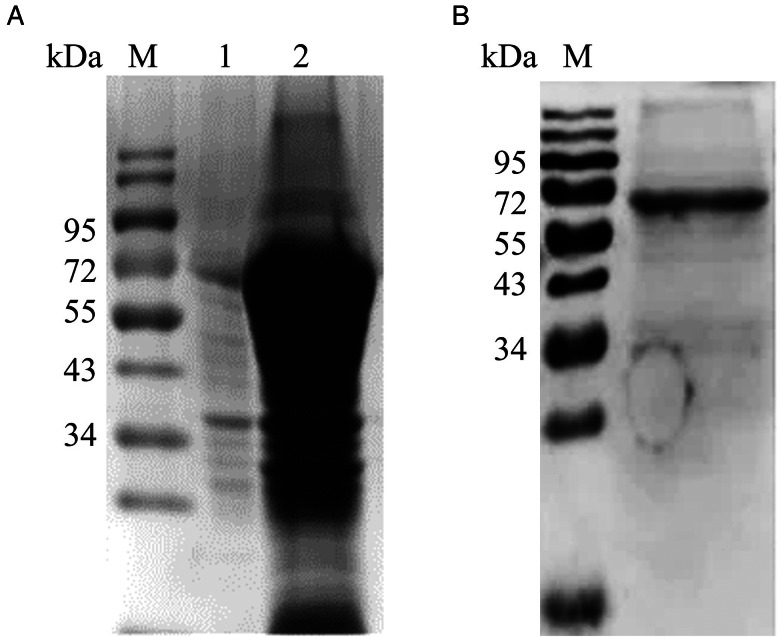
Expression of recombinant protein *Et*PGK. (A) Expression form of recombinant protein. M: protein molecular weight marker; 1: the precipitated; 2: the supernatant. (B) Purification of recombinant protein.

We successfully expressed *Et*PGK in *E. coli* BL21 as a GST-tagged fusion protein. SDS-PAGE showed that r*Et*PGK was expressed mainly in the inclusion bodies (Fig. 2A). Then we purified recombinant protein by cutting gel purification. The molecular mass of the r*Et*PGK fused to the GST-tag (26 KDa) was found to be approximately 68 kDa (Fig. 2B).

### *Et*PGK transcription and translation at different developmental stages of the DS strains

**Figure 3.**
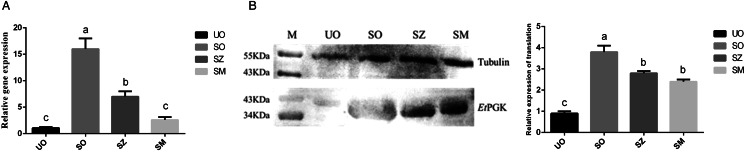
Transcription and translation levels of *Et*PGK in different developmental stages of *E. tenella.* (A) Transcription levels of *Et*PGK. (B) Translation levels of *Et*PGK. UO, unsporulated oocyst; SO, sporulated oocyst; SZ, sporozoite; SM, second generation merozoite. a, b and c indicate significant differences (*P* < 0.05) between groups.

Using 18s rRNA as an internal reference gene, qRT-PCR was used to detect mRNA transcription levels of *Et*PGK at different developmental stages of *E. tenella* DS strains. The results showed that the mRNA transcription levels of *Et*PGK was highest in SO, followed by SZ, and lowest in UO and SM (Fig. 3A). The translation levels of *Et*PGK were examined by western blot using *α*-tubulin as a control. According to Fig. 3B, the translation level of *Et*PGK was higher in SO than other 3 stages, and lowest in UO.

### Differences in transcription and translation levels of *Et*PGK between sensitive and resistant strains

**Figure 4.**
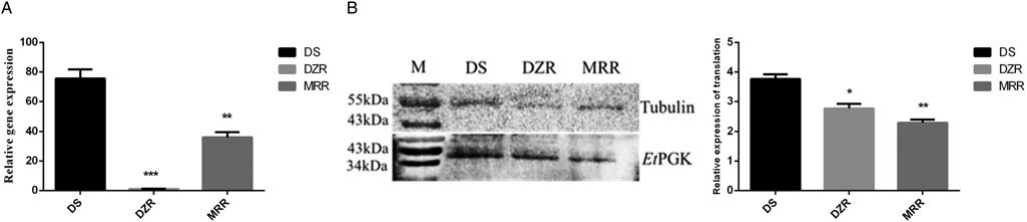
Expression levels of *Et*PGK in drug-sensitive (DS), diclazuril-resistant (DZR) and maduramicin-resistant (MRR) strains. (A) Transcription levels of *Et*PGK. (B) Translation levels of *Et*PGK. *P < 0.05; **P < 0.01; ***P < 0.001.

The mRNA transcription levels of *Et*PGK in DS, DZR and MRR strains of *E. tenella* were detected by qRT-PCR. Using 18S rRNA as an internal reference gene, the transcription levels of *Et*PGK were significantly downregulated in both resistant strains compared with DS strain (Fig. 4A). Whole proteins of *E. tenalla* of DS, DZR and MRR strains were collected and analysed by western blot using *α*-tubulin as a control. The results showed that translation level of *Et*PGK was significantly downregulated in the DZR and MRR strains (Fig. 4B).

**Figure 5.**
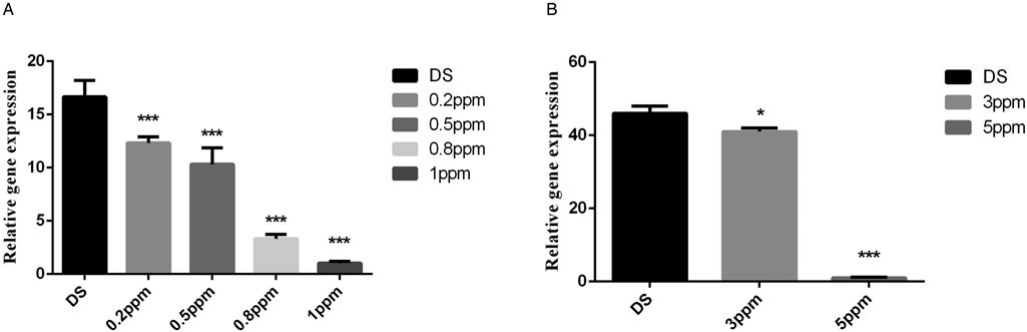
Transcription levels of *Et*PGK in drug-resistant strains at different concentrations. ppm: mg kg^−1^; (A) different concentrations of diclazuril; (B) different concentrations of maduramicin. *P < 0.05; ***P < 0.001.

It was also found that the mRNA transcription levels of *Et*PGK decreased with increasing drug concentrations of maduramicin and diclazuril. The transcription levels of different concentrations of DZR and MRR strains were significantly downregulated compared with the DS strain (Fig. 5).

**Figure 6.**
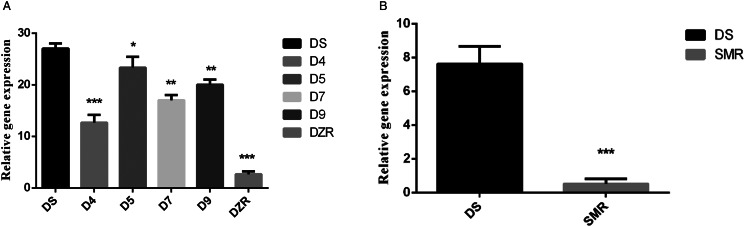
*Et*PGK transcript levels in other drug-resistant strains. (A) Transcription levels of *Et*PGK in diclazuril-resistant strains obtained from the field; D4–D9, 4 field isolated diclazuril-resistant strains. (B) Transcription levels of *Et*PGK in salinomycin-resistant (SMR) strain. *P < 0.05; **P < 0.01; ***P < 0.001.

We analysed mRNA transcription level of *Et*PGK in the field isolated diclazuril-resistant strains (D4, D5, D7 and D9) using qRT-PCR. The result showed that the transcription level of *Et*PGK was also downregulated in the field diclazuril-resistant strains compared with DS strain (Fig. 6A). However, they still have a gap compared with the laboratory-induced DZR strain that is completely resistant to 1.2 ppm diclazuril.

Using qRT-PCR, we also compared the transcription levels of *Et*PGK in the SO of DS and SMR strains. It was found that the transcription level of *Et*PGK in the SMR strain was significantly downregulated compared with the DS strain (Fig. 6B).

### Immunofluorescence localization of *Et*PGK

**Figure 7.**
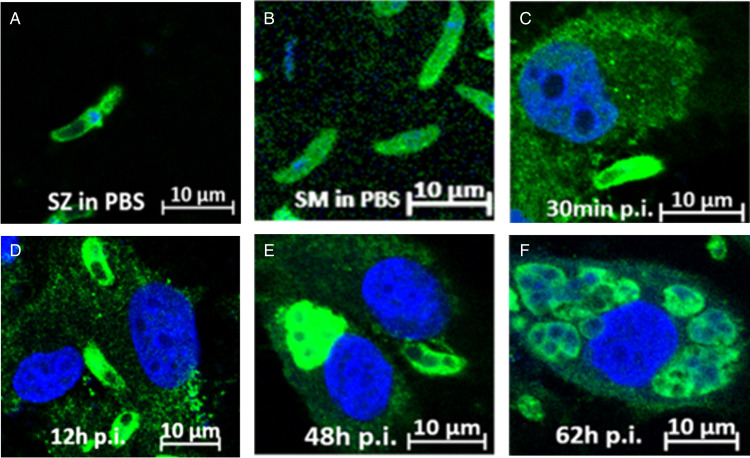
Distribution and localization of *Et*PGK at different development stages. (A) Sporozoites (SZ) in PBS; (B) second-generation merozoites (SM) in PBS; (C) sporozoites at 12 h p.i; (D) immature schizonts (iSc) at 48 h p.i; (E) iScat 62 h p.i.

The distribution of *Et*PGK at different developmental stages of *E. tenella* was analysed by indirect immunofluorescence localization. It was observed that *Et*PGK was mainly located on the surface and in the cytoplasm of the SZ with the exception of the refractile body (Fig. 7A) and SM (Fig. 7B). During the invasion of DF-1 cells by SZ to develop into schizonts, *Et*PGK was distributed on the surface and cytoplasm of schizonts, and the fluorescence intensity was enhanced (Fig. 7C–F).

### Inhibition of *E. tenella* SZ invasion by antibodies against r*Et*PGK

**Figure 8.**
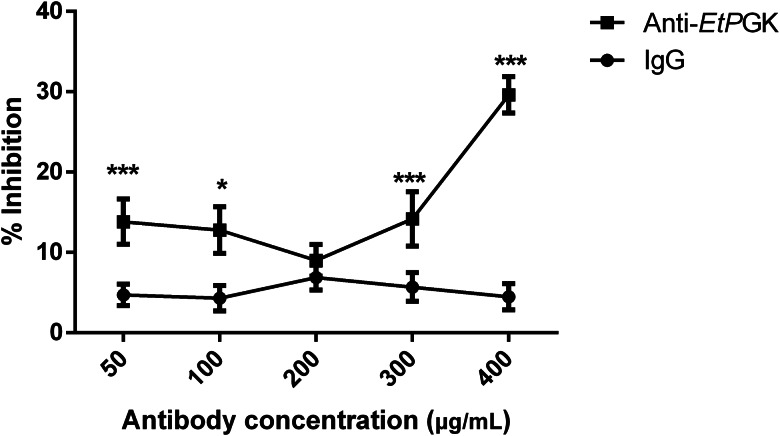
Effect of anti-r*Et*PGK polyclonal antibody on sporozoite invasion.

Invasion inhibition assay was used to determine the effect of rabbit anti-r*Et*PGK polyclonal antibody on the invasion of SZ of DS strain into DF-1 cells. It was found that the inhibition rate was positively correlated with the antibody concentration, and the inhibition rate of rabbit anti-r*Et*PGK polyclonal antibody at a concentration of 400 *μ*g mL^−1^ reached 30%. Meanwhile, rabbit negative IgG showed no significant inhibition of SZ (Fig. 8).

### Enzyme activity

**Figure 9.**
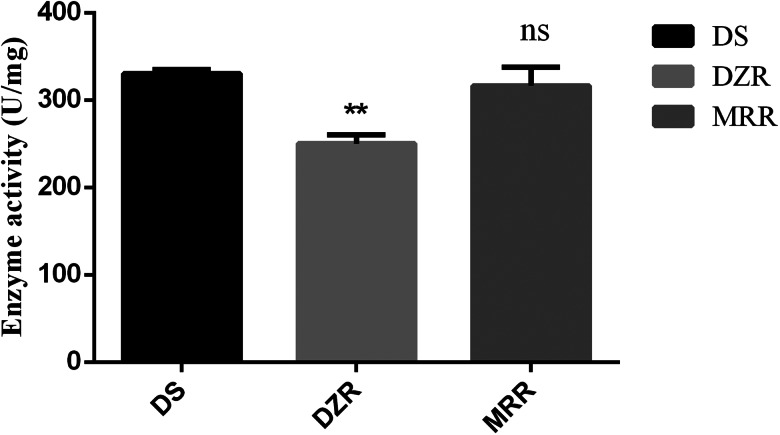
Enzyme activity of *Et*PGK. **P < 0.01.

The enzyme activities of DS, DZR and MRR were analysed using PGK enzyme activity assay kit. The result showed that compared with DS strain, the enzyme activity of *Et*PGK was significantly reduced in the SO of DZR strain. There was no significant difference in *Et*PGK enzyme activity between MRR and DS strain (Fig. 9).

## Discussion

PGK is a glycolytic enzyme that is highly conserved in various organisms and catalyses one of the 2 ATP-generating reactions in the glycolysis pathway. PGK was classified as a moonlighting protein because it is also involved in several different functions separate and distinct from energy metabolism, including pathogenesis, interaction with nucleic acids, tumorigenesis and progression, cell death and viral replication (Rojas-Pirela *et al*., 2020). Our results showed that the transcription and translation levels of *Et*PGK were highest in SO and gradually decreased in SZ and SM. Compared with the other stage, the SO was produced in a relatively oxygen-rich environment *in vitro*. Therefore, we speculated that the change in the expression level of *Et*PGK may be related to changes in the environment, such as temperature and oxygen content. Li *et al*. (2019) found that PGK was widely expressed in most tissues of *Litopenaeus vannamei*, with the highest expression levels in the muscle, where oxygen demand is the highest, followed by the heart and brain. The lowest expression levels were in the small intestine. Some studies have found that PGK contains a Per-Arnt-Sim (PAS) domain. PAS domain-containing proteins are major participants in the adaptation of prokaryotes and eukaryotes to environmental stimuli and can act as input modules to sense oxygen, redox potential and other stimuli (Taylor and Zhulin, 1999; Adhikari *et al*., 2019).

We also found that PGK was distributed on the surface and in the cytoplasm of *E. tenella* through indirect immunofluorescence localization. As a multifunctional protein, its distribution in different parts of the parasite may play different functions. PGK in *Schistosoma japonicum* (*Sj*PGK) was found to be distributed not only in the tegument but also in other tissues. Using qRT-PCR, *Sj*PGK was found to be expressed at all developmental stages studied (Hong *et al*., 2015). Based on its distribution on the parasite surface, we speculated that *Et*PGK might be related to the invasion of *E. tenella* into host cells. Invasion inhibition assays found that SZ incubated with rabbit anti-r*Et*PGK polyclonal antibody serum had a reduced ability to invade DF-1 cells. We hypothesized that *Et*PGK plays an important role in *E. tenella* invasion of cells. Studies have found that PGK is a major outer surface protein of group B streptococci, and PGK can bind to the host's *α*-actinin to facilitate streptococcal invasion of host epithelial cells (Burnham *et al*., 2005). As a glycolytic enzyme, *Et*PGK may provide energy for SZ and SM to invade intestinal epithelial cells. Hong *et al*. (2003) found that miracidia in the eggs of *Clonorchis sinensis* may accumulate PGK to generate the energy required for post-hatching movement. In addition, we speculated whether *Et*PGK would be secreted extracellularly by the parasite to perform its function. We verified by secretion experiments that *Et*PGK was not a secreted protein (data not shown).

Chicken coccidia, such as *Plasmodium* and *Toxoplasma*, are apicomplexan protozoan parasites. These exclusively intracellular parasites develop in a hypoxic or anaerobic environment. Therefore, some genes involved in the tricarboxylic acid cycle and *β*-oxidation pathway were lost in the process of evolution (Jacot *et al*., 2016). Studies found that the mitochondria of apicomplexan protozoan parasites lack type I NADH dehydrogenase and the enzymes and transporters required for fatty acid *β*-oxidation (Mogi and Kita, 2010; Danne *et al*., 2013). Therefore, we speculated that glycolysis would be the main way in which the parasite would obtain energy when the parasite is in the host. Previous studies showed that the transcription and translation levels of enolase 2, glyceraldehyde-3-phosphate dehydrogenase and malate dehydrogenase of *E. tenella* in glycolysis pathway in drug-resistant strains were significantly higher than those in the DS strain, as verified by the results of RNA-seq (Chen *et al*., 2018; Huang *et al*., 2022; Yu *et al*., 2023). A study of drug resistance of *Plasmodium* also found that changes in protein expression levels were closely related to the emergence of drug resistance. Birnbaum *et al*. (2020) found that decreased expression of *Pf*kelch13 protein and its interaction protein around the food vesicle of *Plasmodium* resulted in a decrease in haemoglobin endocytosis and digestion of the parasite. This reduced the activation of artemisinin and made the parasite resistant to artemisinin. Therefore, we hypothesized that the differential expression of *Et*PGK, a key enzyme in the glycolysis pathway, in drug-resistant strains may be closely related to the generation of drug resistance of *E. tenella*.

qRT-PCR and western blot showed that compared with the DS strain of *E. tenella*, the transcription and translation levels of *Et*PGK were downregulated in the 2 drug-resistant strains and were negatively correlated with the drug concentrations of diclazuril and maduramicin. Enzyme activity assays showed that *Et*PGK activity was significantly downregulated in the DZR strain compared with the DS strain. Gene mutations may cause changes in expression level or enzyme activity. Our laboratory has previously performed whole-genome sequencing of DS strains and different diclazuril-resistant strains, and screened for differential SNPs of diclazuril-resistant strains compared to DS strains (data not published). However, we found that *Et*PGK was not screened for gene mutations in the DZR strains. The downregulation of *Et*PGK enzyme activity in the DZR strain may be caused by decreased expression level and other reasons, which requires further research. Western blot revealed that the protein level of *Et*PGK was significantly reduced in the MRR strain. Maduramicin kills parasites mainly by interrupting their normal intracellular ion balance and influencing Na^+^/K^+^-ATPase activity (Chapman *et al*., 2010). This may lead to changes in the content of metal ions such as Na^+^ and K^+^ in the MRR strain, thereby affecting enzyme activity. Lou *et al*. (2024) found that both K^+^ and Na^+^ have a significant effect on the activity of synthetic 7*α*-hydroxysteroid dehydrogenase. In addition, studies have shown that K^+^, Na^+^, Ca^2+^ and Mg^2+^ can promote the activity of esterolytic enzymes in cow rumen (Lee *et al*., 2021). We speculated that Mg^2+^ in the enzyme activity assay kit reaction solution might also have affected *Et*PGK activity. Compared with the DS strain, the mRNA transcription level of *Et*PGK in diclazuril-resistant strains isolated from the field was significantly downregulated. However, there was still a gap between the DZR strain that was induced in the laboratory and completely resistant to 1.2 ppm diclazuril. This may be because although field DZR strains have drug resistance, they are not completely resistant. Differences in their sensitivity to drugs also lead to differences in *E*tPGK expression levels. The study on *Leishmania tropica* found PGK to be differentially expressed in antimony-resistant field isolates (Kazemi-Rad *et al*., 2013). PGK was found to be downregulated in cisplatin-resistant cell lines of oral squamous cell carcinoma, and a study of kidney renal clear cell carcinoma found that PGK enhanced sorafenib resistance and tumorigenesis by regulating glycolysis, which is a promising drug target (Nakatani *et al*., 2005; He *et al*., 2024).

Previous studies found that the upregulated expression of key enzymes of the glycolytic pathway in drug-resistant strains may provide energy for the parasite to antagonize drugs. However, our study found that *Et*PGK was downregulated in drug-resistant strains, which is interesting. Studies have found that the upregulation of mitochondrial PGK by the ROS-TBC1D15 pathway promoted neuronal death after oxygen-glucose deprivation/reoxygenation (OGD/R) injury, while knocking down PGK led to a reduction in OGD/R-induced neuronal death (Chen *et al*., 2024). Other studies showed that overexpression of PGK in H157 lung squamous cell carcinoma cells destroyed the stability of uPAR mRNA and reduced cell surface uPAR expression and uPAR-mediated signalling, mitosis, cell adhesion and migration, and other cellular functions (Shetty *et al*., 2010). Liu *et al*. (2023) found that PGK aggravated cerebral ischaemia-reperfusion injury (CIRI) by inhibiting the Nrf2/ARE signalling pathway and that inhibiting PGK attenuated CIRI by activating the Nrf2/ARE pathway to reduce the release of inflammatory and oxidative factors in astrocytes. Therefore, we speculated that *Et*PGK might antagonize the effects of drugs on *E. tenella* by participating in other pathways, such as downregulating inflammatory pathways, inducing antioxidant pathways, or affecting the expression of parasite surface proteins. However, the specific mechanism of the relationship between *Et*PGK and the development of drug resistance in *E. tenella* requires further studies.

In addition to participating in the glycolytic pathway to provide energy for parasite activities, *Et*PGK may help *E. tenella* adapt to environmental changes，participate in parasites invasion of host cells and development of drug resistance. Compared with DS strain, the downregulated expression of *Et*PGK in resistant strains of *E. tenella* is different from the previous studies of some resistance-related genes. The reasons will be worth exploring in the future. With the development of gene editing in *Eimeria*, we will verify whether *Et*PGK is involved in the development of *E. tenella* drug resistance by overexpression of *Et*PGK in resistant strains or knockdown of *Et*PGK in DS strains. It will not only clarify the relationship between *Et*PGK and the emergence of drug resistance, but also help us to further understand the biological characteristics of *Et*PGK in *E. tenella*.

## Conclusions

In this preliminary study on the function and characterization of *Et*PGK, we found that *Et*PGK was differentially expressed at different developmental stages of *E. tenella*, with the highest expression level at the SO stage. *Et*PGK was located on the surface of the parasites in addition to the cytoplasm of *E. tenella*. Compared with the DS strain, the expression levels of *Et*PGK in the DZR and MRR strains were significantly downregulated. The transcription levels of *Et*PGK were also significantly lower in the field DZR and SMR strains than in the DS strain. The enzymatic activity of *Et*PGK was lower in the DZR strain than in the DS strain. These results suggest that *Et*PGK may play multiple roles in addition to glycolysis. Moreover, *Et*PGK might be involved in the development of drug resistance of *E. tenella* to anti-coccidial drugs.

## Data Availability

All relevant data are within the paper. Sequence data are available on the NCBI GenBank database.
